# *ABSCISIC ACID INSENSITIVE3* Is Involved in Cold Response and Freezing Tolerance Regulation in *Physcomitrella patens*

**DOI:** 10.3389/fpls.2017.01599

**Published:** 2017-09-12

**Authors:** Tinghong Tan, Yanni Sun, Xingji Peng, Guochun Wu, Fang Bao, Yikun He, Huapeng Zhou, Honghui Lin

**Affiliations:** ^1^Key Laboratory of Bio-resource and Eco-environment of Ministry of Education, College of Life Sciences, Sichuan University Chengdu, China; ^2^School of Life Sciences, Capital Normal University Beijing, China

**Keywords:** *Physcomitrella patens*, cold acclimation, freezing tolerance, ABI3, ABA, photosynthetic protein complexes

## Abstract

**Synopsis**

This work demonstrates that PpABI3 contributes to freezing tolerance regulation in Physcomitrella patens.

Transcription factor ABSCISIC ACID INSENSITIVE3 (ABI3) is known to play a major role in regulating seed dormancy, germination, seedling development as well as stress responses. ABI3 is conserved among land plants; however, its roles in non-seed plants under stress conditions have not been well characterized. In this study, we report that ABI3 is involved in freezing tolerance regulation during cold acclimation at least in part through ABA signaling pathway in moss *Physcomitrella patens* (*P. patens*). Deletion of *PpABI3* (Δ*abi3-1*) compromises the induction of genes related to cold response and antioxidative protection, resulting in reduced accumulation of cryoprotectants and antioxidants. In addition, photosystem II (PSII) activity is repressed in Δ*abi3-1* during cold acclimation partially due to alternations of photosynthetic protein complexes compositions. The gametophyte of Δ*abi3-1* displays severe growth inhibition and developmental deficiency under low temperature condition, while two independent complementary lines display phenotypes similar to that of wild-type *P. patens* (WT). Furthermore, the freezing tolerance of Δ*abi3-1* was significantly affected by deletion of *PpABI3*. These data revealed that *PpABI3* plays an important role in low temperature response and freezing tolerance in *P. patens*.

## Introduction

Nearly two-thirds of the world's plants grow in regions below 0°C (Beck et al., 2004; Beike et al., 2015). Freezing stress adversely affects plant growth and crop productivity globally while plants have evolved mechanisms to tolerate freezing stress through cold acclimation. In this way, plants can efficiently activate their cellular signal transduction and metabolisms strategies to acquire enhanced freezing tolerance (Miura and Furumoto, 2013). Previous studies have documented that cold acclimation is a complex process involving many biochemical and physiological changes in plant cells, including alterations of lipid compositions, accumulation of antioxidants and reduction of photoinhibition through non-photochemical quenching (NPQ) (Thomashow, 1999; Beike et al., 2015). During cold acclimation, expression profile of cold-responsive genes (*COR*) is reprogrammed to activate cold responsive signaling and these *CORs* are coordinated with multiple signaling pathways to enhance freezing tolerance in plants (Chinnusamy et al., 2007; Jeon and Kim, 2013; Miura and Furumoto, 2013).

ABSCISIC ACID INSENSITIVE3 (ABI3) is a plant-specific B3 domain-containing transcription factor (TF), which is conserved in higher plant (Romanel et al., 2009). In angiosperm plants, ABI3 participates in seed development and maturation (Monke et al., 2012; Delmas et al., 2013). In addition, ABI3 also plays essential regulatory roles in plastids development, vegetative growth, flowering time regulation and abiotic stress responses (Parcy et al., 1997; Rohde et al., 2000; Frank et al., 2005; Khandelwal et al., 2010). Transcriptomic analysis revealed that ABI3 appears to affect overlapping sets of stress-responsive genes mediated by CBF1/DREB1A, a key TF regulating freezing tolerance in *Arabidopsis* (Tamminen et al., 2001). Over-expression of *ABI3* gene enhances freezing tolerance in response to low temperature in *Arabidopsis* (Tamminen et al., 2001).

*P. patens* is a representative ancestor of land plant which highly tolerates drought, salt, osmotic and cold stress (Frank et al., 2005; Takezawa et al., 2015). Data showed that *PpABI3A* enhances ABA-induced *Em-GUS* expression in *P. patens* in a manner similar to that in angiosperms (Marella et al., 2006). *PpABI3s* are required for *P. patens* vegetative tissue to tolerate desiccation via regulating multiple ABA-responsive genes, which might participate in water deficit responsive modulation (Khandelwal et al., 2010; Yotsui et al., 2016). PpABI3 regulates ABA-responsive genes through ABRE element in their promoter. Specifically, RY motif in *Em* genes is also involved in PpABI3-mediated transcription regulation (Sakata et al., 2010). Furthermore, PpABI3 might regulate set of ABA-responsive gene transcription via the ACTT-core element in coordination with nuclear factor Y (NF-Y) complex (Yotsui et al., 2013). In fact, one of the three homologs of *PpABI3* could partially rescue the phenotype of *abi3-6* mutant of *Arabidopsis* (Marella et al., 2006). These results indicate that ABA signaling and *ABI3* are conserved in angiosperm plants and bryophytes. The whole-genome transcriptomic and proteomic data suggested that cold acclimation in *P. patens* might be similar to that in higher plants (e.g., *Arabidopsis*). However, whether *PpABI3* plays a regulatory role in cold acclimation and freezing tolerance of *P. patens* is not clear.

In this study, we report that *PpABI3* is involved in regulating cold response and freezing tolerance in *P. patens. PpABI3* deletion mutant Δ*abi3-1*, in which all the three *PpABI3* homologs were deleted, displays severe growth inhibition and developmental deficiency of gametophytes under low temperature condition, while complementary expression three gene of *PpABI3* could rescue these phenotype and display similar to that of WT. In addition, deletion of *PpABI3* compromises the induction of genes related to cold response and reduce the accumulation of antioxidants and cryoprotectants. Furthermore, photosystem II activity is significantly repressed in Δ*abi3-1* during cold acclimation and the mutant exhibits lower freezing tolerance despite after cold acclimation. Together, all the results in this study demonstrated that *PpABI3* is involved in regulating cold acclimation and freezing tolerance in *P. patens*.

## Results

### Disruption of *PpABI3* inhibits gametophyte growth of *P. patens* under cold condition

**Figure 1:**
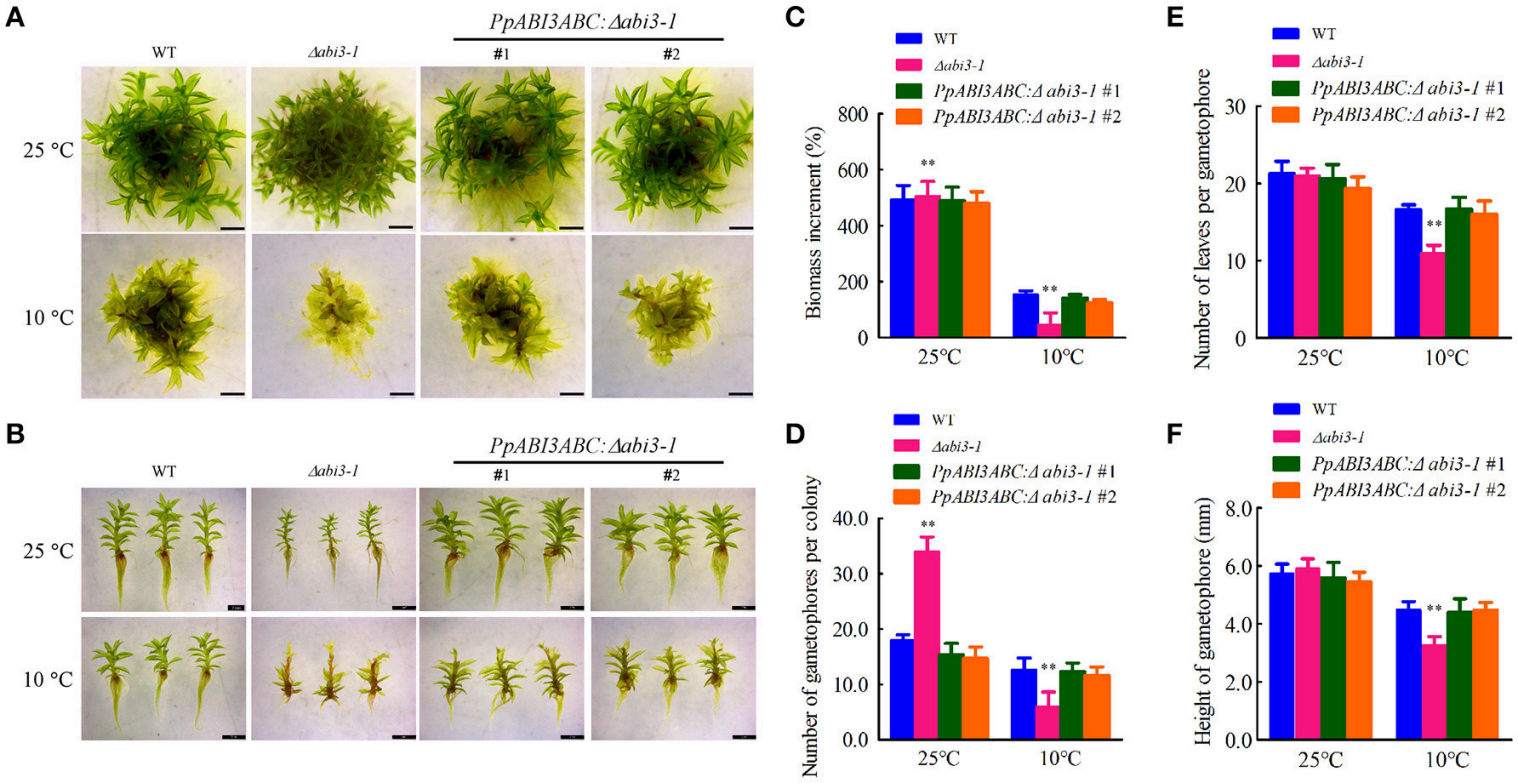
Deletion of *PpABI3* inhibits growth of *P. patens* under cold condition. **(A)** Analysis of gametophyte growth. Seven-day-old tissues of WT, Δ*abi3-1* and two complementary lines were inoculated on fresh BCD medium, and then grown under growth temperature (25°C) or lower temperature (10°C) for 4 weeks before photographs were taken. **(B)** Representative images of gametophores of each strains. Gametophores of wild type, Δ*abi3-1*and two complementary lines incubated under 25°C or 10°C were collected to show their different phenotypes. Bar = 2 mm for all panels. **(C)** Analysis of biomass increment in **(A)**. Fresh weight of each strain was determined for biomass increment analysis in relation to the starting inoculated biomass. Error bars represent SD (*n* = 3) and Two-way ANOVA was used to determine the statistical significance (^**^P < 0.01). **(D)** Number of gametophores that emerged on the colonies of each strain. The colonies were incubated under 25°C or 10°C for 4 weeks and gametophores that emerged were counted. Error bars indicate mean ± SD (*n* = 6) and Two-way ANOVA was used to determine the statistical significance (^**^P < 0.01). **(E)** Number of leaves that generated per gametophore of each strain. The colonies were incubated under 25°C or 10°C for 4 weeks and representative gametophores were collected for estimation of the leaves amount. Error bars indicate mean ± SD (*n* = 10) and Two-way ANOVA was used to determine the statistical significance (^**^P < 0.01). **(F)** Mean height of gametophores analysis for the wild type, Δ*abi3-1* and *PpABI3ABC*:Δ*abi3-1* lines. Error bars indicate mean ± SD (*n* > 60) and Two-way ANOVA was used to determine the statistical significance (^**^P < 0.01).

*P. patens* genome encodes three *ABI3* genes, *PpABI3A, PpABI3B*, and *PpABI3C*, which share higher identity in the conserved basic domains (Marella et al., 2006; Figures S1A,B; Table S2). To investigate the possible regulatory role of *PpABI3* in cold response, expression of *PpABI3* genes was first determined in WT with or without cold acclimation. We found that *PpABI3A* and *PpABI3B* were significantly induced in protonema incubated under 10°C for 2 weeks, while expression of *PpABI3C* was only slightly induced by low temperature (Figure S1C). These data indicated that all the *PpABI3* genes might positively regulate cold response in *P. patens*. The effect of *PpABI3* disruption on gametophyte growth and development under low temperature condition was then investigated. *PpABI3* deletion mutant line Δ*abi3-1*, in which all three *PpABI3* genes were deleted by sequential gene targeting, was generated (Khandelwal et al., 2010; Figures S2A,B). Seven-day-old protonematal tissues of WT and mutant strains were then cultured at growth temperature (25°C) or low temperature (10°C) for 4 weeks. Compared to the growth condition, the growth of WT and Δ*abi3-1* were both repressed under 10°C. The biomass of Δ*abi3-1* was significantly lower than that of wild type during cold stress (Figures 1A,C). Meanwhile, Δ*abi3-1* generated less gametophore during cold incubation than that of WT, although this activity was even higher in the mutant when grown at 25°C (Figures 1A,D). Interestingly, we found that transcription of two genes, *PpSHI1* (Pp1s373_11V6) and *PpSHI2* (Pp1s19_109V6) which function in auxin biosynthesis (Eklund et al., 2010), was higher in Δ*abi3-1* compared to that in WT when grown at 25°C, which might explain why the number of gametophore per colony in the mutant is higher than in WT, and suggest a possible role of *PpABI3* in auxin biosynthesis (Figure S3). In addition, the cormus of Δ*abi3-1* was shorter and possessed fewer leaves after low temperature incubation (Figures 1B,E,F; Figure S4).

We have also generated the complementary lines (*PpABI3ABC:*Δ*abi3-1*) by introducing all the three *PpABI3* genes into Δ*abi3-1* background and confirmed the complementary lines by RT-PCR assay (Figure S2C). Data showed that the growth inhibition in Δ*abi3-1* was rescued by *PpABI3A, PpABI3B* and *PpABI3C* in the complementary lines (Figures 1A–F). The elevated electrolyte leakage (EL) in Δ*abi3-1* under low temperature condition was also decreased to a level similar to WT in both complementary lines (Figure S2D). These results together demonstrated that deletion of *PpABI3* represses the gametophyte growth of *P. patens* under low temperature condition and *PpABI3* might be involved in regulating growth and development in *P. patens*.

### *PpABI3* participates in regulating cryoprotectants and antioxidants accumulation during cold acclimation

**Figure 2:**
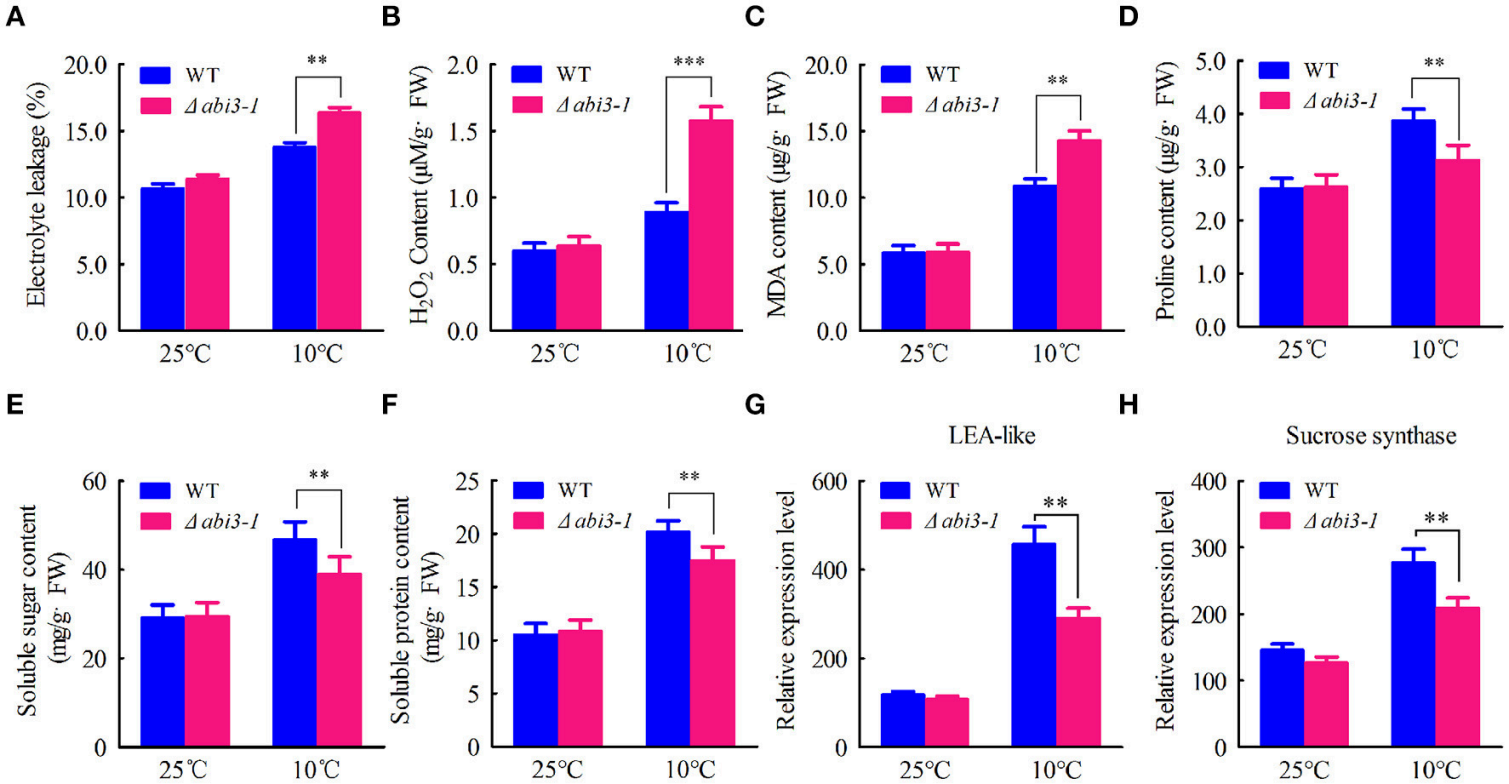
*PpABI3* participates in low temperature response of *P. patens*. **(A–C)** Analysis of cell injury and oxidative damage. Tissues of WT and Δ*abi3-1* with or without cold acclimation under 10°C were collected to determine electrolyte leakage (EL) rate **(A)**, hydrogen peroxide (H_2_O_2_) content **(B)** and malonyldialdehyde (MDA) content **(C)**. **(D–F)** Analysis the accumulation of cryoprotectants. Tissues of WT and Δ*abi3-1* with or without cold acclimation were collected to estimate proline content **(D)**, total soluble sugar content **(E)** and total soluble protein content **(F)**. Error bars represent SD (*n* = 3) and Two-way ANOVA was used to determine the statistical significance (^**^P < 0.01). **(G,H)** Quantitative RT-PCR to analyse the expression of a late embryogenesis abundant (LEA)-like protein-coding gene (Pp1s267_21V6.1) and a sucrose synthase-coding gene (Pp1s93_98V6.1). *PpACTIN5* (Pp1S381_21V6) was used as internal control. Error bars represent SD (*n* = 3) and Two-way ANOVA was used to determine the statistical significance (^**^P < 0.01; ^***^P < 0.001).

Since *PpABI3* plays a role in cold response, we then hypothesized that they might contribute to cold acclimation regulation. Cold stress imposes cell injury and oxidative damages to plants (Mahajan and Tuteja, 2005; Demidchik et al., 2014; Hossain et al., 2015). To determine whether *PpABI3* is involved in regulating cell injury and redox homeostasis during cold acclimation, WT and Δ*abi3-1* mutant were subjected to 25°C or 10°C for 2 weeks, then EL and ROS accumulation were determined. Data showed that both EL and ROS were induced in WT and Δ*abi3-1* after incubation under 10°C (Figures 2A,B). However, the accumulation of EL and H_2_O_2_ in Δ*abi3-1* were significantly higher than that in WT (Figures 2A,B), indicating that cold stress disrupted cellular redox homeostasis and caused severely cell injury in Δ*abi3-1*. We further detected the accumulation of MDA, a marker of lipid oxidation in response to environmental stresses (Davey et al., 2005). The content of MDA was also higher in Δ*abi3-1* compared to that of WT under low temperature condition (Figure 2C). These results indicated that *PpABI3* is involved in low temperature response in *P. patens*. In addition, accumulations of cryoprotectants including proline, soluble sugar and soluble protein, all of which could contribute to cold acclimation, were significantly lower in Δ*abi3-1* than that in WT (Figures 2D–F). Consistently, we found that the expression of LEA-like protein and sucrose synthase which function in plant cold response (Beike et al., 2015) were also much lower in Δ*abi3-1* under low temperature condition (Figures 2G,H). These results demonstrated that *PpABI3* plays a regulatory role in redox homeostasis and cryoprotectants accumulation during cold acclimation.

**Figure 3:**
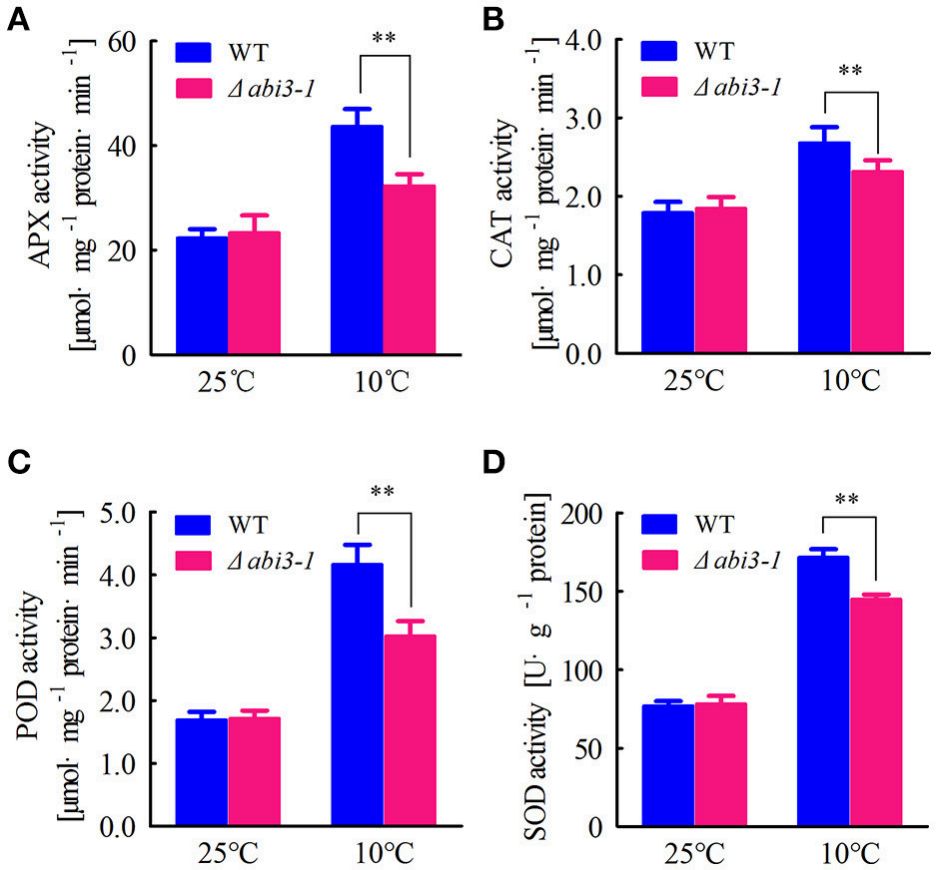
*PpABI3* is involved in regulating enzymatic antioxidative signaling. **(A–D)** Antioxidant enzymatic activities analysis. Protonema after incubation under standard growth temperature (25°C) or low temperature (10°C) for 2 weeks were harvested to analyze ascorbate peroxidase (APX) activities **(A)**, catalase (CAT) activities **(B)**, glutathione peroxidase (POD) activities **(C)** and superoxide dismutase (SOD) **(D)**. Error bars represent SD (*n* = 3) and two-way ANOVA was used to determine the statistical significance (^**^P < 0.01). Experimental details are found in Materials and Methods.

**Figure 4:**
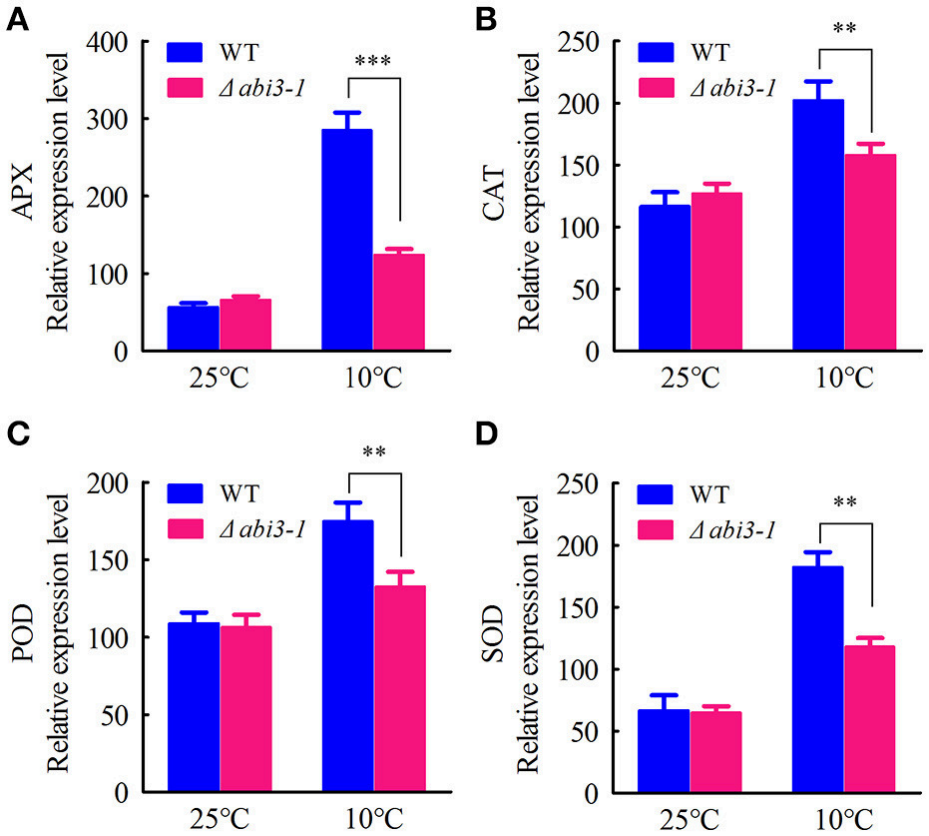
Expression of antioxidant enzymes mediated by *PpABI3* during cold acclimation. **(A–D)** The transcriptional levels of genes encoding ascorbate peroxidase (APX: Pp1S277_34V6), catalase (CAT: Pp1S422_8V6), glutathione peroxidase (POD: Pp1S98_2V6) and superoxide dismutase (SOD: Pp1S22_320V6) were analyzed by quantitative RT-PCR in wild-type and Δ*abi3-1* with or without cold acclimation. Error bars represent SD (*n* = 3), and two-way ANOVA was used to determine the statistical significance (^**^P < 0.01; ^***^P < 0.001).

Cellular antioxidative signaling will be induced to protect cells from oxidative damage by scavenging ROS in plants during cold acclimation (Blokhina et al., 2003; Gill and Tuteja, 2010). We then determined the enzymatic activity of ascorbate peroxidase (APX), catalase (CAT), peroxidase (POD) as well as superoxide dismutase (SOD) in WT and Δ*abi3-1* under control and cold conditions. Data showed that the activity of APX, CAT, POD and SOD were relatively increased in both WT and Δ*abi3-1* during cold acclimation (Figures 3A–D). However, the increase amplitudes of the four enzymes in Δ*abi3-1* were significantly lower than in WT (Figures 3A–D). These results suggest that *PpABI3* contributes to redox homeostasis regulation during cold acclimation in *P. paten*. We further found that transcription level of genes encoding antioxidant enzymes, *PpAPX* (Pp1s277_34V6), *PpCAT* (Pp1s422_8V6), *PpPOD* (Pp1s98_2V6), and *PpSOD* (Pp1s22_320V6), in Δ*abi3-1* were much lower than that in WT during cold acclimation (Figures 4A–D). These results were consistent with the lower antioxidant enzymatic activity in Δ*abi3-1* during cold acclimation (Figures 3A–D).

### *PpABI3* participates in regulating expression of multiple genes during cold acclimation

**Figure 5:**
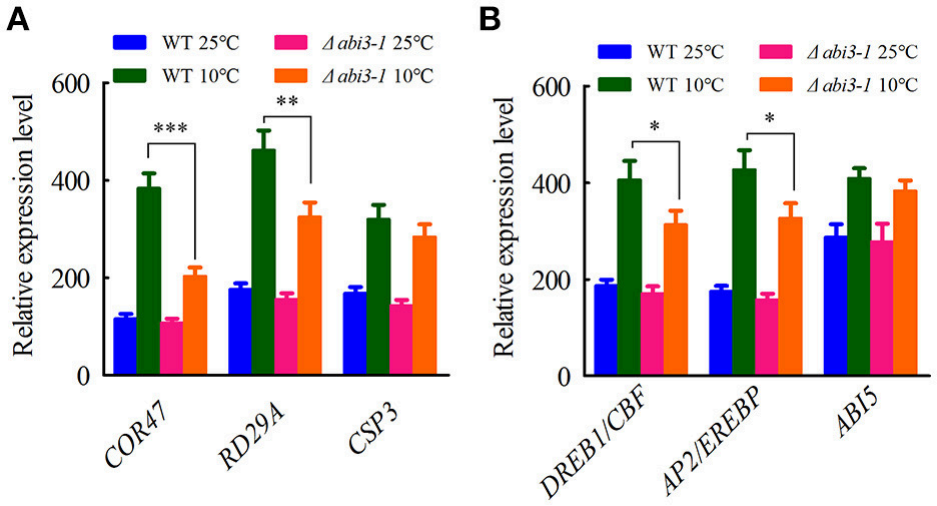
Cold-responsive genes are regulated by *PpABI3* during cold acclimation. **(A)** Relative transcription of three cold responsive genes. The transcriptional levels of three genes: *PpCOR47* (Dehydrin-coding gene: Pp1s442_22V6.2), *PpRD29A* (Desiccation-responsive protein 29A: PP1S203_40V6.1) and *PpCSP3* (Cold shock protein: Pp1s103_65V6.1) were analyzed by quantitative RT-PCR. **(B)** Relative transcription of representative transcription factors. The transcriptional levels of three cold responsive transcription factors: *DREBP1/CBF* (DRE/CRT binding factor: Pp1s60_228V6.1), *AP2/EREBP* (APETALA2/ethylene-responsive element binding protein: Pp1s373_18V6.1) and *ABI5* (*ABSCISIC ACID INSENSITIVE5*: Pp1s49_161V6) were analyzed by quantitative RT-PCR. Error bars represent SD (*n* = 3), and two-way ANOVA was used to determine the statistical significance (^*^P < 0.05; ^**^P < 0.01; ^***^P < 0.001).

To investigate the mechanism by which *PpABI3* regulates cold acclimation, we next analyzed the contributions of *PpABI3* in regulating cold-responsive genes (*COR*) during cold acclimation. We found that the cold-induction target genes such as *PpCOR47* (Pp1s442_22V6.2), *PpRD29A* (Pp1s203_40V6.1), and *PpCSP3* (PP1S103_65V6.1) were drastically repressed in Δ*abi3-1* compared to WT (Figure 5A), which correlates with the growth inhibition of Δ*abi3-1* under low temperature. Interestingly, two transcription factors, *PpDREB1/CBF* (Pp1s60_228V6.1) and *PpAP2/EREBP* (Pp1s373_18V6.1), which also contributed to cold acclimation regulation (Beike et al., 2015), were induced significantly in WT during cold acclimation, while their accumulation in Δ*abi3-1* was also partially repressed (Figure 5B), suggesting that *PpABI3* might regulate cold acclimation in part through *PpDREB1/CBF* and *PpAP2/EREBP*. We also determined the transcription of *PpABI5* (Pp1s49_161V6) in WT and Δ*abi3-1 P. patens*. Although ABI3 and ABI5 are intensely related in higher plants (Lim et al., 2013), we did not observe any detectable difference of *PpABI5* transcription between WT and Δ*abi3-1* under both control and cold conditions (Figure 5B). Together, these results suggested that *PpABI3* is involved in cold acclimation by regulating *COR* genes expression in *P. patens*.

**Figure 6:**
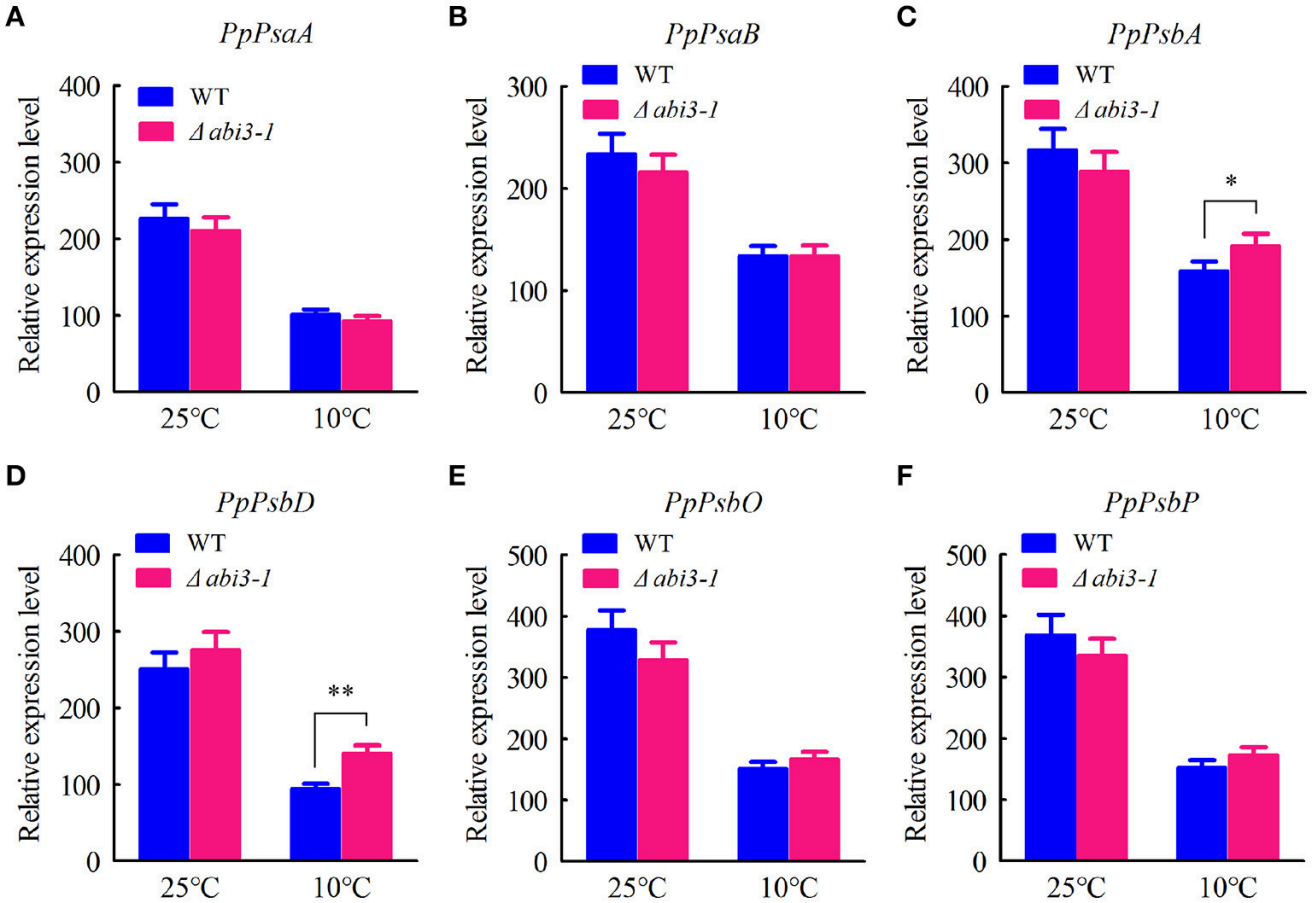
Expression pattern of photosynthetic genes. **(A–F)** The transcriptional levels of genes related to photosystem manganese-stabilizing including *PpPsaA* (PhpapaCp039), *PpPsaB* (PhpapaCp040), *PpPsbA* (PhpapaCp046), *PpPsbD* (PhpapaCp044), *PpPsbO* (Pp1s60_65V6.1) and *PpPsbP* (Pp1s63_71V6.1) were determined by quantitative RT-PCR assay. Error bars represent SD (*n* = 3), and two-way ANOVA was used to determine the statistical significance (^*^P < 0.05; ^**^P < 0.01).

Interestingly, expression of photosynthetic genes including *PpPsaA* (PhpapaCp039), *PpPsaB* (PhpapaCp040), *PpPsbA* (PhpapaCp046), *PpPsbD* (PhpapaCp044), *PpPsbO* (Pp1s60_65V6.1) as well as *PpPsbP* (Pp1s63_71V6.1) were also determined, aiming to analyze the possible effect of *PpABI3* disruption on photosynthesis related transcriptome regulation. The results showed that all these candidate genes expressed at similar level under control condition in wild-type and mutant lines (Figures 6A–F). However, expression of these genes decreased during cold acclimation in both WT and Δ*abi3-1*, indicating that low temperature might repress photosynthetic activity through restricting expression of these core components of photosynthetic apparatus (Figures 6A–F). Interestingly, we found that expression of *PpPsbA* and *PpPsbD* was relatively higher in Δ*abi3-1* than that in WT although other genes showed similar expression level in both lines (Figures 6C,D), indicating that transcription of several photosynthetic proteins might be affected due to *PpABI3* deletion during cold acclimation. Together, we proposed that *PpABI3* might participate in photosynthesis regulation at least in part by modulating expression of photosynthetic genes during cold acclimation. On the other hand, we still need to determine whether photosynthesis and photosynthetic apparatus were affected in Δ*abi3-1*.

### Disruption of *PpABI3* reduces PSII activity in *P. patens* during cold acclimation

**Figure 7:**
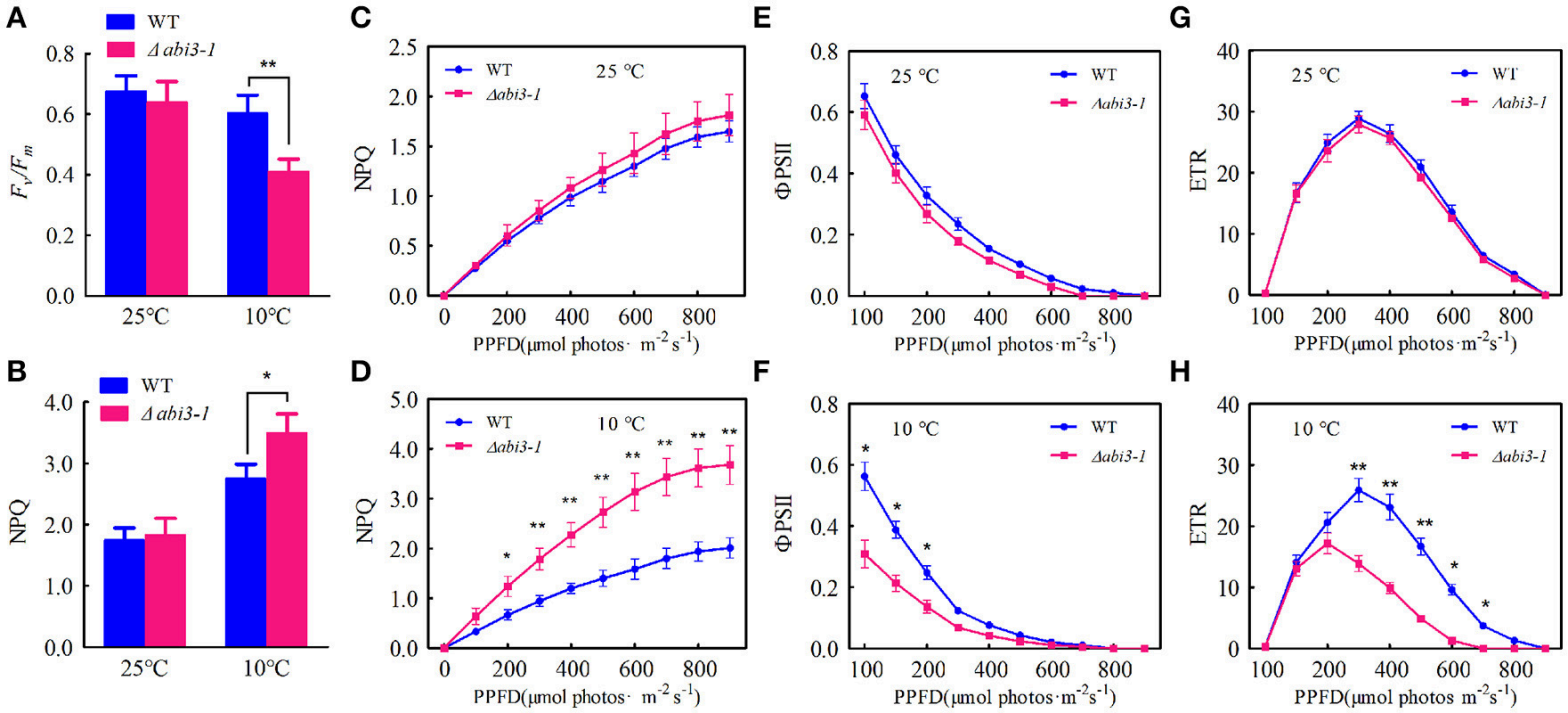
Deletion of *PpABI3* reduces PSII activity during cold acclimation. **(A–H)** Chlorophyll fluorescence analysis. Two-week-old protonema of WT and Δ*abi3-1* with or with cold acclimation were collected to determine the maximum photochemical efficiency of PSII (*F*_*v*_*/F*_*m*_) **(A)**, steady-state non-photochemical quenching (NPQ) **(B)**, light-response curves of non-photochemical quenching (NPQ) **(C,D)**, the efficiency of PSII quantum yield (ΦPSII) **(E,F)** and PSII electron transport rate (ETR) **(G,H)**. Error bars represent SD (*n* = 3), two-way ANOVA was used to determine the statistical significance (^*^P < 0.05; ^**^P < 0.01). Experimental details are found in the Materials and Methods.

Low temperature significantly represses photosynthetic capacity in plants (Hurry et al., 2000; Paul and Foyer, 2001; Ensminger et al., 2006). We then analyzed the putative function of *PpABI3* in regulating photosynthesis during cold acclimation. The maximum photochemical efficiency of PSII [variable fluorescence (*F*_*v*_)/maximun fluorescence (*F*_*m*_)] were determined. Data showed that, *F*_*v*_*/F*_*m*_ reduced in both WT and Δ*abi3-1* after 2-week cold acclimation, and it was significantly lower in Δ*abi3-1* compared to WT (Figure 7A; Figure S5). In contrast, the non-photochemical quenching (NPQ) were induced during cold incubation, whereas this upregulation was dramatically higher in Δ*abi3-1* (Figures 7B–D). These results indicate that Δ*abi3-1* might suffer severer photoinhibition during cold acclimation. We further determined the light-responsive efficiency of PSII quantum yield (ΦPSII) in WT and Δ*abi3-1*. Under growth condition, increase of light intensity represses the efficiency of ΦPSII, and the light curve of ΦPSII in Δ*abi3-1* was similar to that in WT (Figure 7E). After incubation in 10°C for 2 weeks, ΦPSII decreased rapidly along with light intensity increasing in both WT and Δ*abi3-1*, however, the decline amplitude of ΦPSII in Δ*abi3-1* was more remarkable (Figure 7F). Furthermore, there exists no obvious difference of the maximum value of ETR between WT and Δ*abi3-1* under control condition. However, The maximum value of ETR in Δ*abi3-1* was significantly lower than that in WT, and the decrease of ETR in Δ*abi3-1* was more rapidly than that in WT under low temperature condition (Figures 7G,H), indicating a restricted electron transport, which might result from photoinhibition. In fact, long term low temperature incubation caused photoinhibition was consistent to growth inhibition of Δ*abi3-1*. These results suggest that *PpABI3* contributes to maintaining the PSII activity in response to cold stress.

### Assembly of PSII is perturbed in Δ*abi3-1* during cold acclimation

**Figure 8:**
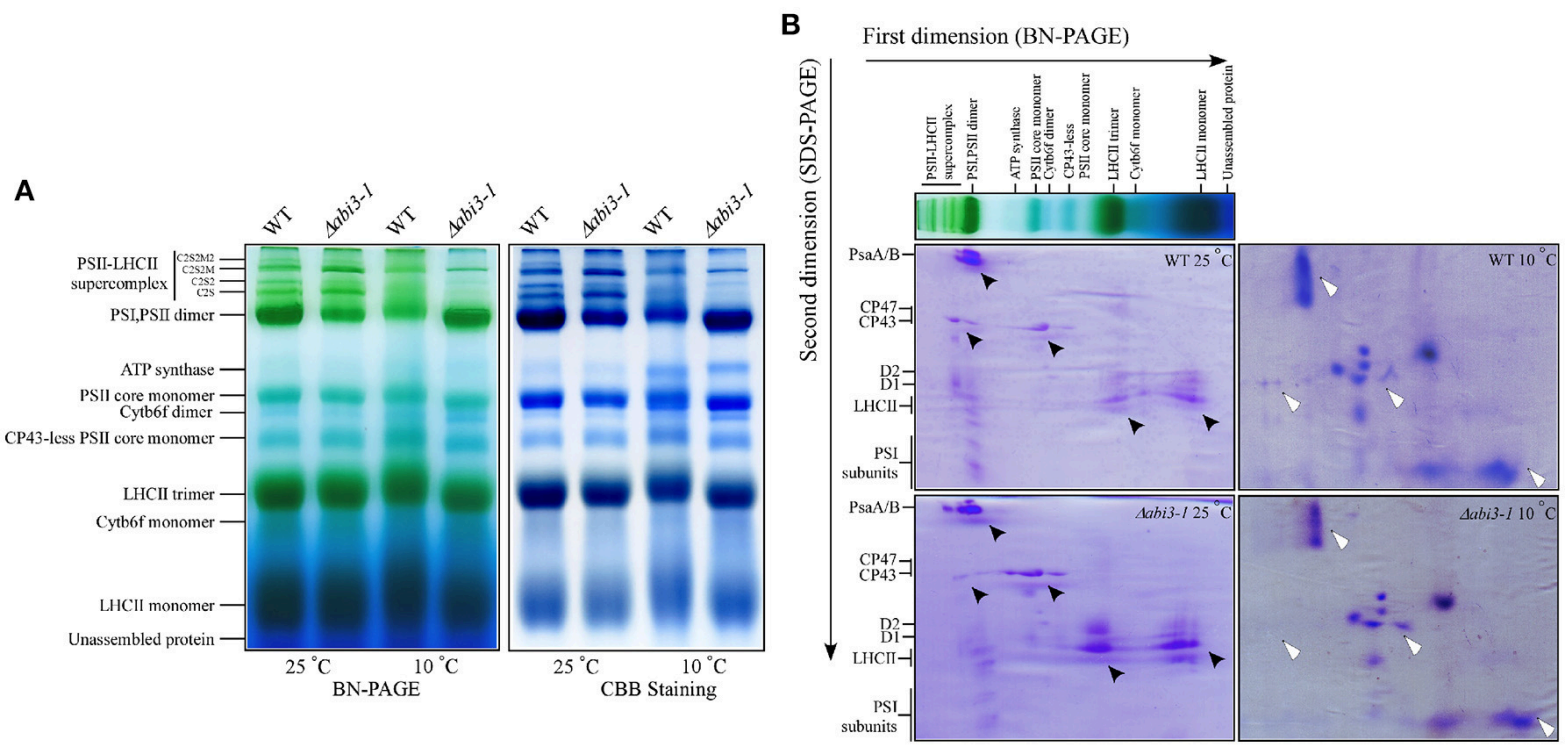
Deletion of *PpABI3* affects assembly of PSII during cold acclimation. **(A)** Blue Native (BN)-gel electrophoresis analysis of thylakoid membrane protein complexes. Thylakoid membranes were isolated from WT and Δ*abi3-1* with or without cold acclimation, then solubilized with 2% DM and separated by BN-PAGE. A sample with an equal amount of chlorophyll (25 μg) was loaded in each lane. The BN gel (left) was further stained with Coomassie Brilliant blue (CBB; right). **(B)** Two-dimensional (2D) separation [BN-gel follows SDS-PAGE] of thylakoid protein complexes. Complexes in BN-gel were subsequently separated by SDS-urea-PAGE and stained with CBB to show their constituent subunits. Protein distribution differences between WT and Δ*abi3-1* were indicated by triangle symbol (black, standard condition; white, after cold acclimation).

PSII activity defects might be caused by alternation of the compositions of photosynthetic apparatus (Baker, 1991; Sato et al., 1995; Lokstein et al., 2002). To analyze the possible role of *PpABI3* in regulating compositions of photosynthetic protein complexes during cold acclimation, thylakoid membranes of both WT and Δ*abi3-1* were solubilized with n-dodecyl β-D-maltoside (DM) and subjected to Blue Native (BN)-gel electrophoretic assay. Data revealed that during cold acclimation, there exist significant differences in compositions of PSII-LHCII supercomplexes and PSI/PSII dimers between WT and Δ*abi3-1* (Figure 8A). Compared to growth condition, PSII-LHCII supercomplexes were reduced in both WT and Δ*abi3-1*. However, accumulation of this supercomplexe in Δ*abi3-1* was significantly lower than that in WT (Figure 8A). In addition, the accumulation of PSI/PSII dimers in Δ*abi3-1* was slightly higher than that in WT during cold acclimation, which might result from reduction of PSII supercomplexes (Figure 8A). Interestingly, protein level of ATP synthase was elevated in both wild-type and mutant lines during cold acclimation (Figure 8A), indicating a regulatory role of photosynthetic phosphorylation during cold acclimation. Two-dimesional analysis (SDS-PAGE follows BN gel electrophoretic assay) showed that the distribution of subunits in PSII core proteins such as D1 and D2 in Δ*abi3-1* were obviously different from that in WT, although the accumulation of other protein subunits was more or less similar between control and cold conditions (Figure 8B). Together, these results suggested that disruption of *PpABI3* significantly perturbs assembly of PSII during cold acclimation and results in decreased photosynthetic capacity.

### *PpABI3* is involved in ABA-dependent freezing tolerance in *P. patens*

**Figure 9:**
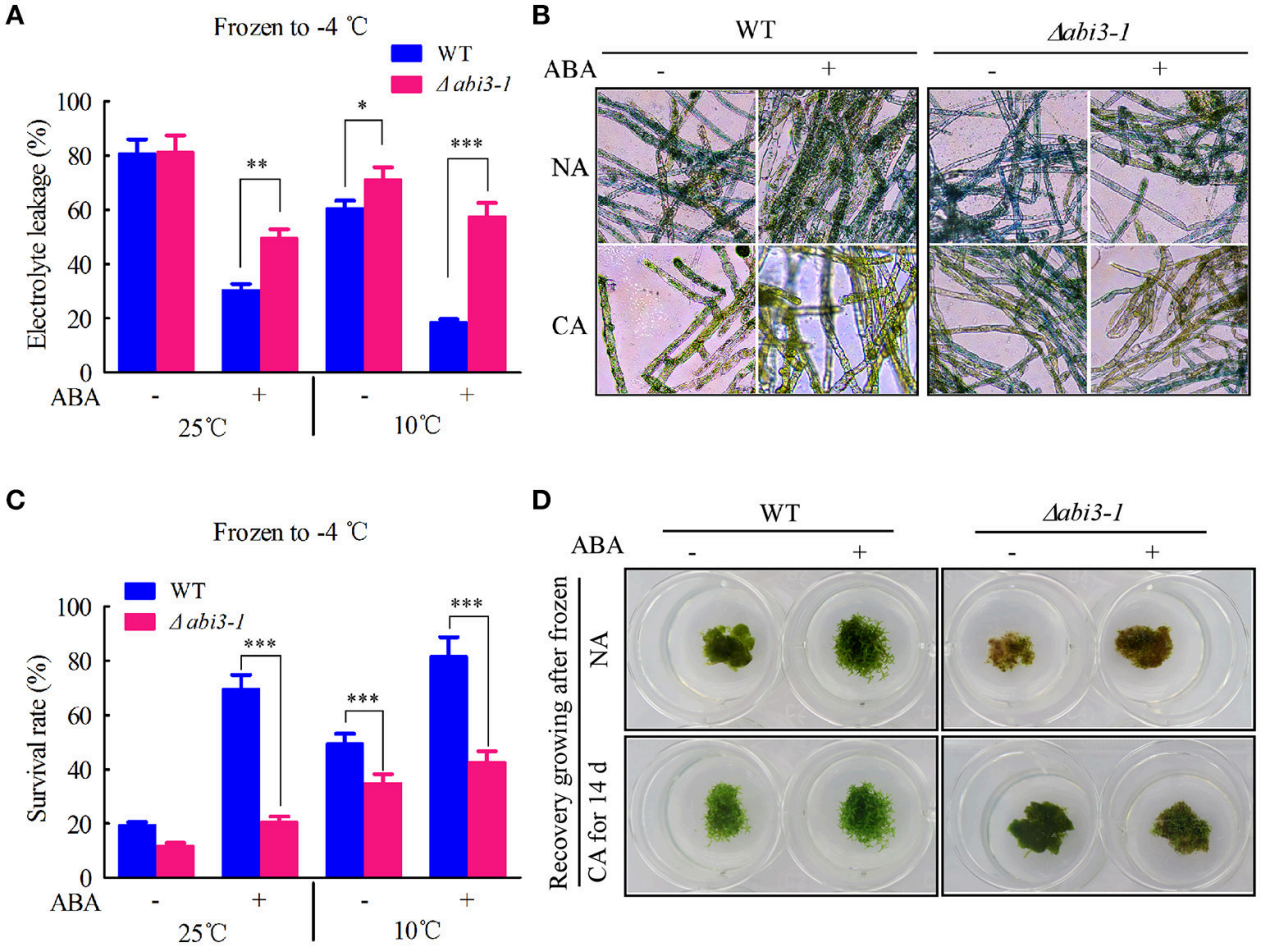
*PpABI3* is involved in ABA-dependent freezing tolerance regulation. **(A)** Electrolyte leakage rate analysis. Tissues of WT and Δ*abi3-1* with or without cold acclimation were treated or not with 1 μM ABA, then frozen to −4°C. After thawing, electrolyte leakage was measured to estimate cell injury. Error bars represent SD (*n* = 3), and two-way ANOVA was used to determine the statistical significance (^*^P < 0.05; ^**^P < 0.01; ^***^P < 0.001). **(B)** Cell death rate estimation. Frozen-thawed cells were stained with 0.5% Evans Blue to analysis the cell death of WT and Δ*abi3-1*. **(C)** Survival rate analysis. Tissues of WT and Δ*abi3-1* with or without cold acclimation were treated or not with 1 μM ABA, then frozen to −4°C, after thawing the cells survival rate were estimated according to Evans Blue staining in **(B)**. Error bars represent SD (*n* = 3), and two-way ANOVA was used to determine the statistical significance (^***^P < 0.001). **(D)** Recovery growing after frozen to −4°C. Frozen-thawed protonematal tissues of WT and Δ*abi3-1* were transferred onto fresh BCD medium and regrown under standard condition for 7 days to estimate the recovery growth capacity.

Cold acclimation is essential for freezing tolerance in plants (Guy, 1990; Thomashow, 1999). Plants acquire enhanced freezing tolerance after cold acclimation (Shinozaki and Yamaguchi-Shinozaki, 2000; Kaplan et al., 2004; Chinnusamy et al., 2007). Since cold acclimation related cellular signaling and metabolism were significantly disturbed in Δ*abi3-1*, which accumulated less cryoprotectants and antioxidants with disrupted redox homeostasis during cold acclimation, we proposed that *PpABI3* disruption might impair freezing tolerance in *P. patens*. To confirm this hypothesis, both WT and Δ*abi3-1* were left untreated or treated under 10°C for 2 weeks (NA or CA) and then frozen to −4°C. After thawing, freezing tolerance was determined by quantifying EL from injured cells. As expected, the freezing tolerance was enhanced both in WT and Δ*abi3-1* after cold acclimation, respectively (Figure 9A). However, EL in Δ*abi3-1* was significantly higher than that in WT (Figure 9A), indicating a lower freezing tolerance of Δ*abi3-1*. These data suggested that *PpABI3* positively modulates freezing tolerance via regulating cold acclimation in *P. patens*.

Previous study has revealed that cold acclimation contributes to freezing tolerance in *P. patens* at least in part depends on ABA signaling (Bhyan et al., 2012). We further investigated the regulatory role of *PpABI3* in ABA signaling associated cold acclimation and subsequent freezing tolerance in *P. patens*. Results showed that exogenous ABA treatment significantly enhanced freezing tolerance in WT, while Δ*abi3-1* failed to acquire additional freezing tolerance after ABA treatment with or without cold acclimation (Figure 9A). To confirm that the EL is associated with actual cell death resulted from freezing, cell survival and cell death were analyzed in WT and Δ*abi3-1* by cells staining with Evans Blue. Data revealed that cold acclimation repressed freezing induced cell death in both WT and Δ*abi3-1*, however, disruption of *PpABI3* led to higher level of cell death and which is correlated with the EL during freezing stress (Figures 9B,C). Consistently, exogenous ABA cannot enhance freezing tolerance due to *PpABI3* deletion, since Δ*abi3-1* displayed similar cell death rate whether with or without ABA treatment (Figures 9B,C). Subsequently, the frozen-thawed *P. patens* of WT and Δ*abi3-1* were left for further recovery analysis. We found that the recovery growth was consistent with the survival rate (Figures 9C,D), indicating that *PpABI3* and ABA signaling also contribute to *P. patens* recovery growth after freezing stress is released. Together, these data suggested that *PpABI3* is involved in ABA-dependent freezing tolerance regulation in *P. patens* during cold acclimation.

## Discussion

To elucidate the involvement of *PpABI3* in the cold acclimation and freezing tolerance in *P. patens, PpABI3* deletion mutant Δ*abi3-1* was used for investigation. To avoid artificial results, we also generated complementary lines by introducing all the three *PpABI3* genes into Δ*abi3-1* background. We found that the growth inhibition of Δ*abi3-1* under low temperature condition was remarkable, and due to *PpABI3* loss-of-function, Δ*abi3-1* exhibited less freezing tolerance than WT. Disruption of *PpABI3* resulted in lower survival rate and repressed recovery growing after frozen to −4°C. H_2_O_2_ and MDA contents in Δ*abi3-1* were higher than that in WT. In addition, the accumulation of cryoprotectants such as proline in Δ*abi3-1* was lower than that in WT during cold acclimation. Due to disruption of *PpABI3*, activity of ROS scavenging enzymes was significantly lower during cold acclimation. Cold-induction of cold-responsive (*COR*) genes transcription in Δ*abi3-1* was repressed during cold acclimation compared to WT. Photosynthetic genes expression and photosynthetic apparatus compositions were significantly altered in Δ*abi3-1*. These results were consistent to the freezing-sensitive phenotype of Δ*abi3-1* and demonstrated that *PpABI3* is involved in cold acclimation and freezing tolerance regulation in *P. patens*.

ABA contributes to freezing tolerance in *P. patens* during cold acclimation (Bhyan et al., 2012). In this study, we found that exogenous ABA treatment enhanced the freezing tolerance in WT, however, Δ*abi3-1* exhibited no significant difference in freezing tolerance with or without ABA treatment. These results were similar to the previous work, which showed that ABA-insensitive lines have barely increased freezing tolerance after ABA incubation (Bhyan et al., 2012). Together these results, we propose that ABA signaling contributes to cold acclimation and freezing tolerance at least in part through *PpABI3*. In fact, the ACTT-core element (5′-TCCACTTGTC-3′) in the promoter of several ABA-responsive genes is required for PpABI3 transcription regulation (Yotsui et al., 2013). It would be interesting to determine whether those genes targeted by PpABI3 during cold response also contain this element. On the other hand, Δ*abi3-1* also acquired partial freezing tolerance after cold acclimation, because the acclimated Δ*abi3-1* mutant can partially withstand freezing temperatures than the non-acclimated ones, supporting that *PpABI3*-independent pathways exist to regulate cold acclimation. In fact, C-repeat binding factors (CBFs), including CBF1, CBF2, and CBF3, contribute to cold acclimation in *Arabidopsis* (Medina et al., 1999; Gilmour et al., 2004). In addition, we found that induction of TFs such as *PpDREB1/CBF* and *PpAP2/EREBP* was partially inhibited in the Δ*abi3-1* during cold acclimation, suggesting that there might exists a crosstalk between PpABI3 and PpCBFs in cold acclimation. To further determine the function of *PpABI3*, the transcriptomic profile of wild type and Δ*abi3-1* during cold acclimation needs to be investigated in the future studies.

During cold acclimation, plants need to adapt to a long-term of environmental changes (Huner et al., 1998) and overcome energy imbalance caused by adverse environment (Miura and Furumoto, 2013). Energy imbalance might cause excess PSII excitation pressure which leads to photoinhibition or photodamage (Gray et al., 1997; Huner et al., 1998; Miura and Furumoto, 2013; Pinnola et al., 2013). Photoinhibiton reduces photosynthetic activity, resulting in plant growth repression (Takahashi and Badger, 2011). Results in this study showed that PSII activity in Δ*abi3-1* was much lower than that in WT, especially during cold acclimation, which might explain the repressed growth due to *PpABI3* disruption. In addition, the NPQ was much higher under low temperature condition in Δ*abi3-1*, suggesting the Δ*abi3-1* processed higher excess PSII excitation energy which needs to be dissipated (Pinnola et al., 2013). Low temperature-caused excess PSII excitation would lead to down-regulation of PSII activity through dissipation of excess energy (which resulted in increase of NPQ and decrease of ETR) or inactivation of PSII irreversibly and subsequently inhibit photosynthetic activity (which resulted in decrease of ΦPSII) (Oquist and Huner, 2003). Our data showed that both ETR and ΦPSII were decreased in Δ*abi3-1* compared to WT during cold acclimation, suggesting that disruption of *PpABI3* decreased photosynthetic capacity might due to photoinhibition. Moreover, compositions of photosynthetic protein complexes in Δ*abi3-1* were clearly different from that in WT, suggesting that the steady state levels of photosynthetic proteins were altered because of *PpABI3* disruption, which would cause severely impacts on plant photosynthesis during cold acclimation.

Taken together, we identified that *PpABI3* was involved in cold acclimation induced freezing tolerance in *P. patens*. However, we still need to investigate how *PpABI3* modulates cellular ROS homeostasis and photosynthetic protein complexes compositions during cold acclimation. Previous studies have revealed that cold stress causes photoinhibition and negatively regulates plant photosynthesis by affecting the repair process of PSII (Lei et al., 2014; Gururani et al., 2015; Zhang et al., 2016). Since PSII repair needs efficient and appropriate synthesis of a variety of proteins *de novo*, we proposed that TF *PpABI3* might function in such process and contributes to freezing tolerance in *P. patens*. In addition, we should further determine whether redox homeostasis is involved in *PpABI3*-mediated proteins synthesis during cold acclimation.

## Materials and methods

### Plant materials and cold acclimation

Protonema tissues of *P. patens* (Gransden, wild-type), and Δ*abi3-1* were grown axenically on BCD medium containing 0.5% (w/v) glucose, 0.75% (w/v) agar, supplemented with 5 mM ammonium tartrate and cultured in greenhouse at 25°C under 16 h light/8 h darkness with light intensity of 50 μmol m^−2^ s^−1^ (Wang et al., 2014). After 1 week, protonematal tissues were transferred onto ammonium tartrate-free BCD medium for gametophyte growth (Cove et al., 2009). Two-week-old protonema was transferred to 25°C or 10°C under standard growth light condition for low temperature incubation.

### Plasmid construction and transformation

The full coding sequences of *PpABI3A, PpABI3B*, and *PpABI3C* were amplified with the PpABI3A-CF/CR, PpABI3B-CF/CR, and PpABI3C-CF/CR primers, respectively. The resulting fragments were then cloned into the modified pTFH15.3 vector between the *Eco*RV and *Apa*I sites. Primer sequences are found in Table S1. Transformation of *P. patens* protoplasts and isolation of transgenic lines was performed as described (Khandelwal et al., 2010).

### Growth analysis

One-week-old wild-type and Δ*abi3-1 P. patens* generated from 1.5 mL protonema suspension were transferred to 25°C and 10°C under standard growth light. Biomass formation starting at the day of inoculation and ending at week four was estimated by measuring fresh weight of per petri dish tissues. The measured raw data were transformed to a percentage value in relation to the inoculated biomass at the starting point which was defined as 0% and growth rate was calculated.

### ABA treatment and freezing tolerance analyzes

Freezing tolerance was determined by measuring electrolyte leakage (EL) after the protonematal tissues were thawed from equilibrium freezing (−4°C) as previously described (Minami et al., 2003) with slight modifications. Protonema under growth condition or incubated under low temperature (10°C) for 2 weeks were transferred to fresh BCD medium added with 1 μM ABA (Sigma) and incubated at 25°C for 24 h. Tissues were collected in test tubes containing 0.5 mL of sterile distilled water. Put the tubes in a liquid bath of 50% ethylene glycol and kept at −1°C for 10 min. Adding pieces of ice into the liquid bath to initiate freezing, and incubating at −1°C for 1 h. The tubes were then cooled to −4°C at a rate of 2°C per hour. Then, remove liquid bath and keep the test tubes at 4°C in darkness until completely thawed. Add 2 mL distilled water to each thawed sample, and incubate them at room temperature for 2 h with gentle shaking in the dark, and then EL of the frozen-thawed tissues was measured. For recovery growing, small pieces of the protonematal tissues after frozen-thawing were inoculated onto a fresh BCD medium and cultured at standard growth condition.

### Electrolyte leakage measurements and evans blue staining

Electrolyte leakage (EL) was measured as previous description (Komatsu et al., 2013). The protonematal tissues thawed after frozen to −4°C were stained for 1 h in 0.5% (w/v) solution of Evans Blue (Sigma, E2129) and observed using microscopy as described to determine survival rate during freezing stress (Wertman et al., 2012).

### Hydrogen peroxide content assay

The H_2_O_2_ content was analyzed as previously described (Xu et al., 2012). Briefly, approximately 0.5 g protonematal tissues were homogenized in an ice bath with 5 mL 0.1% (w/v) trichloroacetic acid (TCA). The homogenate was centrifuged at 12,000 g for 20 min at 4°C. Then 0.5 mL of the supernatant was added to 0.5 mL 10 mM potassium phosphate buffer (pH 7.0) and 1 mL 1 M KI. The absorbance of the supernatant was read at 390 nm.

### Malonyldialdehyde content assay

Malonyldialdehyde (MDA) content was measured as the method described by Lei et al. (2010) with slight modifications. Approximately 0.2 g of protonematal tissues were homogenized with 5 mL of 5% TCA in an ice bath. The homogenate was centrifuged at 1,000 g for 10 min at 4°C. Aliquots of the supernatant and 0.5% thiobarbituric acid (TBA) in 20% TCA solution were transferred into a new tube. The mixture was incubated in water bath at 100°C for 30 min, then cooled to room temperature and centrifuged at 8,000 g for 5 min. The supernatant was subjected for absorbance reading at A535/A600 using a spectrophotometer. The MDA content was calculated from the subtracted absorbance using the Extinction Coefficient of 155 mM^−1^ cm^−1^.

### Proline content determination

Proline content was estimated using the acid-ninhydrin method according to Bates et al. (1973) and Wang et al. (2008) with modifications. 0.5 g protonematal tissues were homogenized with 5 mL of 3% (w/v) sulphosalicylic acid and filtered to obtain the clear filtrate. Glacial acetic acid and ninhydrin reagent (1 mL each) were added to 1 mL of the filtrate. The mixture was heated in an oven at 100°C for 1 h and the reaction was terminated in an ice bath. The reaction mixture was extracted with 4 mL toluene and absorption of the chromophore was read at 520 nm. Proline content was calculated using a standard curve constructed with L-proline standards.

### Antioxidant enzymatic activity analysis

For the enzymatic activity assays, 0.3 g protonema were ground with 3 mL ice-cold 25 mM Hepes buffer (pH 7.8) containing 0.2 mM EDTA, 2 mM ascorbate and 2% polyvinylpyrrolidone (PVP). The homogenates were centrifuged at 12,000 g for 20 min at 4°C and the supernatants were used for the enzymatic activity determination (Zhang et al., 2015). The superoxide dismutase (SOD, EC 1.15.1.1) activity was assayed with NBT following the method of Stewart and Bewley (1980). The catalase (CAT, EC 1.11.1.6) activity was measured as the decline in the absorbance at 240 nm with the method of Patra and Mishra (1979). Peroxidase (POD, EC 1.11.1.7) activity was determined using guaiacol as substrate according to Hammerschmidt et al. (1982). The ascorbate peroxidase (APX, EC 1.11.1.11) activity was measured at 290 nm (Nakano and Asada, 1981).

### Quantitative RT-PCR assays

Total RNA was extracted as previous description (Xi et al., 2010). DNaseI (Sigma) was added to remove DNA in extracted RNA. Two micrograms of RNA was reverse transcribed by ThermoScript TM RT-PCR System (Invitrogen). The cDNA was amplified by using SYBR Premix Ex Taq (TaKaRa). *PpACTIN5* was used as internal control. Genomic DNA was extracted from 1 week-old protonema as previously described (Allen et al., 2006). The absence of *PpABI3A, PpABI3B*, and *PpABI3C* were confirmed in Δ*abi3-1* by PCR using DNA as template. RT-PCR assay was performed with cDNA synthesized from total RNA from wild-type and Δ*abi3-1* to further confirm the expression of *PpABI3*. All primers are described in Table S1.

### Chlorophyll fluorescence analysis

Chlorophyll fluorescence was analyzed using chlorophyll fluorometer (IMAG-MINI PAM-2000; Heinz Walz, Effeltrich) with red (630 nm) pulse modulated measuring light at room temperature. *P. patens* were dark-adapted for 20 min before measurements. Values of *F*_*v*_*/F*_*m*_ (maximum PSII photochemical efficiency) and nonphotochemical quenching (NPQ) were averaged from an approximately rectangular interested area (Gould et al., 2010). Color-indexed images of the samples showing chlorophyll fluorescence parameters were captured using the Imaging PAM software. To analyze the PSII quantum yield (ΦPSII) and the electron transport rate (ETR), *P. patens* were illuminated at the following light intensities: 0, 84, 155, 187, 307, 369, 461, 789, and 909 μmol photons m^−2^ s^−1^ (Lu et al., 2011).

### Thylakoid membranes isolation and BN-PAGE assays

Thylakoid membranes were isolated as described by Lu et al. (2011) with minor modifications. Samples of *P. patens* were crushed and homogenized with ice-cold extraction solution I [50 mM HEPES-KOH, pH 7.5, 330 mM sorbitol, 2 mM EDTA, 1 mM MgCl_2_, 5 mM ascorbate, 0.05% bovine serum albumin and 10 mM NaF]. Ascorbic acid was added to the buffer immediately before homogenization. The homogenates was filtered through four layers of cheesecloth and centrifuged at 2,000 g for 4 min at 4°C. The precipitation was resuspended in solution II [50 mM HEPES-KOH, pH 7.5, 5 mM sorbitol, 10 mM NaF], then centrifuged at 2,000 g for 4 min at 4°C. The thylakoid pellet was resuspended and centrifuged twice in solution III [50 mM HEPES-KOH, pH 7.5, 100 mM sorbitol, 10 mM MgCl_2_, 10 mM NaF]. The final pellet was resuspended in a small volume of solution III. After chlorophylls were extracted with 80% (v/v) aqueous acetone and quantified (Wellburn, 1994), thylakoids were rapidly frozen in liquid nitrogen and stored at −80°C for further assays.

BN-PAGE assay was performed as described (Malnoe et al., 2014). Thylakoids were solubilized with 2% (w/v) dodecyl β-D-maltoside (Sigma) on ice for 30 min. After centrifugation at 13,000 g for 10 min at 4°C, the supernatant was supplemented with 0.1 vol sample buffer containing 100 mM Bis Tris-HCl, pH 7.0, 500 mM 6-amino-caproicacid, 30% (w/v) glycerol, 5% (w/v) Serva blue G, and subjected to BN-PAGE with a gradient of 5–13.5% Bis-Tris mini separation gel. Electrophoresis was performed at 4°C with cathode buffer (50 mM Tricine, 15 mM Bis-Tris, pH 7.0, and 0.01% Coomassie Brilliant Blue G 250) and anode buffer (50 mM Bis-Tris, pH 7.0) for 25 min at 50 V and then 2 h at 150 V.

### Statistical analysis

Means of at least three biological replicates were measured for each assay. Two-way ANOVA was used for comparison between different treatments. A difference was considered to be statistically significant when *P* < 0.05, *P* < 0.01, or *P* < 0.001, respectively.

## Author contributions

TT, HZ, and HL designed the experiments. TT performed major of the study. YS, XP, GW, and FB provided assistance. TT, HZ, YH, and HL analyzed the data. HZ and TT wrote the manuscript and contributed to discussion.

### Conflict of interest statement

The authors declare that the research was conducted in the absence of any commercial or financial relationships that could be construed as a potential conflict of interest.

## Abbreviations

ABA: abscisic acid
ABI3: ABSCISIC ACID INSENSITIVE3
APX: ascorbate peroxidase
BN-PAGE: blue-native polyacrylamide gel electrophoresis
CAT: catalase
CBB: Coomassie Brilliant Blue
Chl: chlorophyll
COR: cold-responsive genes
EL: electrolyte leakage
ETR: electron transport rate
MDA: malondialdehyde
NF-Y: nuclear factor Y
NPQ: non-photochemical quenching
PAM: pulse-amplitude modulated
POD: peroxidase
PPFD: photosynthetically active photon flux density
ROS: reactive oxygen species
RWC: relative water content
SOD: superoxide dismutase
ΦPSII: PSII quantum yield.
