# Supplementary material for: ABSCISIC ACID INSENSITIVE3 Is Involved in Cold Response and Freezing Tolerance Regulation in Physcomitrella patens
Source: Front Plant Sci. 2017 Sep 12;8:1599. doi: 10.3389/fpls.2017.01599 (PMC5601040; doi:10.3389/fpls.2017.01599)
Supplement: Supplementary file 1 [file Table1.pdf]

Table S1. Primers used in this study

Primer Name	Primer Sequence	Gene ID
PpABI3A-qRT-F	ATGAACACGCGATGGAATCAT	Pp1s7_115V6
PpABI3A-qRT-R	AACTTATCTCCATCAGTCGATGGT	
PpABI3B-qRT-F	AAACAACACAATGGTGAGATG	Pp1s173_143V6.1
PpABI3B-qRT-R	GTGACTTGATGCACTGAAATA	
PpABI3C-qER-F	GGGAGCGCCACCGTGCAACA	Pp1s143_82V6
PpABI3C-qER-R	CCTGATCCCGAGGACTGGTTTCG	
AP2/EREBP-qRT-F	GGAGATGAAGAAGTCGATAGG	Pp1s373_18V6.1
AP2/EREBP-qRT-R	TCGGAAATGAGAATTAGAAGA	
DREB1-qRT-F	CCTAACGGCTCCAAAACT	Pp1s60_228V6.1
DREB1-qRT-R	CTCGCTGAACGAACATCT	
COR47-qRT-F	ACAGAGAGTCCGAAGACAGAGG	Pp1s442_22V6.2
COR47-qRT-R	AGGCAAGGATAAGAGAACAGGG	
RD29A-qRT-F	TTTTCCTTTTGCCCTATTTT	Pp1s203_40V6.1
RD29A-qRT-R	ATTGATCTGGTTGTGTCTCG	
CSP3-qRT-F	GTTGGCGAATGTCTCTGA	Pp1s203_40V6.1
CSP3-qRT-R	ACGAAATGGAAATGTGGT	
SUS-qRT-F	ACAGGAACAAACCCATCCT	Pp1s93_98V6.1
SUS-qRT-R	CCGAGACATCGCTTACAAC	
LEA-qRT-F	ATGCTGCTAAGGATACCACAC	Pp1s267_21V6.1
LEA-qRT-R	ATCAAACACGATAGAAAAACG	
PsaA-qRT-F	TGTGATGGACCTGGAAGAG	Pp1s143_15V6
PsaA-qRT-R	AAACTAAATGCCCAAACGA	
PsaB-qRT-F	TACTCGTCGTATTTGGTTCGGT	Pp1s143_15V6
PsaB-qRT-R	ACTTGGTTTCCATTTTGGTTGT	
PsbA-qRT-F	TCTCTGCTGCTATCATCCCTAC	PhpapaCp046
PsbA-qRT-R	TTCTTCCTCTTGACCAAACTTG	
PsbD-qRT-F	CTTTAATAGGCTTTATGTTGCG	PhpapaCp044
PsbD-qRT-R	AGTTTCTTCAGATTGAGTTGGG	
PsbO-qRT-F	GCTGGAGCAGTAGACACAGAC	Pp1s60_65V6.1
PsbO-qRT-R	ATCAAACGCATAAAGGAAAGA	
PsbP-qRT-F	AAAGGCAGACCCTACTACAA	Pp1s63_71V6.1
PsbP-qRT-R	TATCCTCCACAAACCAATCC	
CAT-qRT-F	GGCTGTGCTAGTCGGTGGT	Pp1s422_8V6
CAT-qRT-R	AAACGGTGACGTTGGGTGT	
APX-qRT-F	AGACGATGAGACGAGGCGG	Pp1s277_34V6
APX-qRT-R	GGAGAGGGAAATGGCAGGC	
POD-qRT-F	GGATTTTGAGATTTTGGCAT	Pp1s98_2V6
POD-qRT-R	TTTGAAGGAGGAGTAGTGGG	
SOD-qRT-F	GGGCTATGTGGCGAATCT	Pp1s22_320V6
SOD-qRT-R	CTACGACCAGCAAGGGGA	
PpABI3A-RT-F	TCTTCCACTTGCAGTCCATT	Pp1s7_115V6

PpABI3A-RT-R	ATCCATCTCGCACTCTAACC	
PpABI3B-RT-F	AGTGGACATAGGGAGATTGG	Pp1s173_143V6
PpABI3B-RT-R	TGTGACATACTTGCCTACGAT	
PpABI3C-RT-F	CATCATTCTCCTCGTCATTG	Pp1s143_82V6
PpABI3C-RT-R	AGATTGGGTGGTTCTTCCTT	
PpABI3A-CF	ACGATATCATGGTGCTCCTATCGAGTGTGGAAG	Pp1s7_115V6
PpABI3A-CR	AGGGGGCCCTCATCCTGCGGGCTCGGTCTTCAC	
PpABI3B-CF	ACGATATCATGGATGGCAATGTGCGAGCTGTGT	Pp1s173_143V6
PpABI3B-CR	AGGGGGCCCTCATCCCACGAGCTCCGCCAATTC	
PpABI3C-CF	ACGATATCATGGTGAACCAAGGCAGCGGGGATT	Pp1s143_82V6
PpABI3C-CR	AGGGGGCCCCTACTCCAGCTCTGTCTTGGGG	
PpACT5-qRT-F	GGATTGCGAAAAGCGAG	Pp1s381_21V6
PpACT5-qRT-R	CCGTTAGAATTGAGCCC	
PpIAA1a-qRT-F	ATGAACGTCAGCGAAGGTTGTAG	Q948Q2
PpIAA1a-qRT-R	CTTGTTGCATGTGATATGCGGACC	
PpIAA1b-qRT-F	GCCTGTTCCGGATGGTGGTGATT	Pp1s184_21V6
PpIAA1b-qRT-R	GCACGTCTCCGACCAGCATCG	
PpTIRB1-qRT-F	CCAGACGAGGTGCTGGAGCATG	Pp1s137_148V6
PpTIRB1-qRT-F	GCAGATCCAGCTCCGTGAGATGC	
PpTIRB2-qRT-F	CTCTGCGCCTTGCCAGCTGTGAT	Pp1s196_87V6
PpTIRB2-qRT-F	CAGTAGGCGCCGGAGCTTGACGC	
PpSHI1-qRT-F	CTCGCACTTTAAGGGACGACG	Pp1s373_11V6
PpSHI1-qRT-R	CTGGTTGAGATGGGTGACCAGAG	
PpSHI2-qRT-F	GTTCCACAAGTTATGGGCCACGG	Pp1s19_109V6
PpSHI2-qRT-R	CAGCGCTTGCGCACGCACTTCC	
PpGA20ox4-qRT-F	CTCAGTCGTTGAAGAATACATGG	Pp1s180_73V6
PpGA20ox4-qRT-R	GCATGCCATGGATAAACGCTGG	
PpGA20ox6-qRT-F	CCTGAAGGATCCTCCAGTCAGCA	Pp1s106_2V6
PpGA20ox6-qRT-R	CGCAAATACTGGCTGAAGCAGCT	
PpGAL1-qRT-F	CTGCTTAGCTTGCCACCGGGTCC	PHYPADRAFT_235432
PpGAL1-qRT-R	ATTCGAATTCTACCTTGACGGT	
PpCPS/KS-qRT-F	GGCTTCCAGCACCTTGATACAG	Pp1s130_5V6
PpCPS/KS-qRT-R	CACTCGCGCTACCCAAGCTGTGT	
PpABI5-qRT-F	GAACGCTGAGCATGAAGACGGT	Pp1s49_161V6
PpABI5-qRT-R	TCCGCTGCTGCCTGCTGCTGTGCG	
