# Supplementary material for: ABSCISIC ACID INSENSITIVE3 Is Involved in Cold Response and Freezing Tolerance Regulation in Physcomitrella patens
Source: Front Plant Sci. 2017 Sep 12;8:1599. doi: 10.3389/fpls.2017.01599 (PMC5601040; doi:10.3389/fpls.2017.01599)
Supplement: Supplementary file 2 [file Table2.pdf]

Table S2. Information of ABI3/VP1 proteins among indicated species

**Species name**	**Protein ID**
A.thaliana	At3g24650
M.truncatula	XP_013448806.1
P.trichocarpa	XP_002303088.2
T. aestivum	CBH32542.1
O.sativa	Os01g68370
P.patens	BAE80314.1
	BAE80315.1
	BAE80317.1
C.reinhardtii	XP_001693653.1
