# Supplementary material for: ABSCISIC ACID INSENSITIVE3 Is Involved in Cold Response and Freezing Tolerance Regulation in Physcomitrella patens
Source: Front Plant Sci. 2017 Sep 12;8:1599. doi: 10.3389/fpls.2017.01599 (PMC5601040; doi:10.3389/fpls.2017.01599)
Supplement: Supplementary file 3 [file Presentation1.pdf]

Figure S1

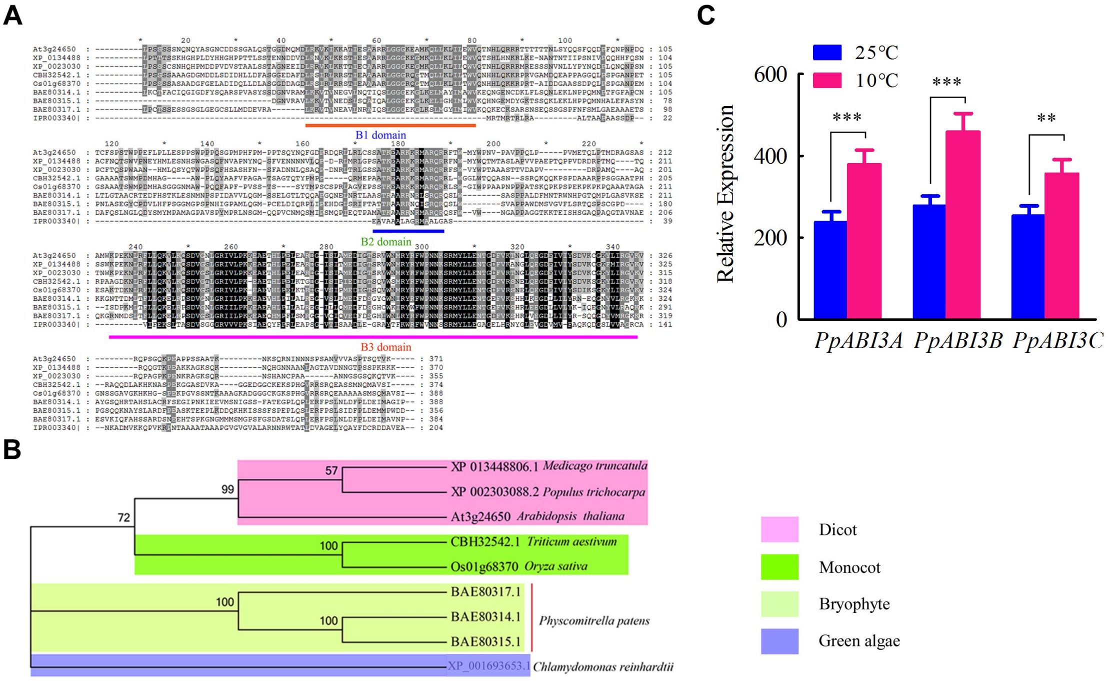

Figure S1. Protein sequences alignment, phylogenetic relationship between *ABI3* genes among indicated species, and induction of *PpABI3* in *P. patens*.

(A). Alignment of amino acid sequences of ABI3/VP1 among *Arabidopsis, Medicago truncatula, Populus trichocarpa, Triticum aestivum, Orysa sativa, P. patens* and *Chlamydomonas reinhardtii*. Identical residues are shaded black and similar residues are shaded grey. The B1, B2 and B3 domains are indicated respectively by the red, blue, or magenta underlining.

(B). Rooted Neighbour-joining tree of ABI3 families based on the B3 domain amino acid sequence of *Chlamydomonas reinhardtii*. Different color code represents the phylogenic position and classification of various *ABI3* genes.

(C). Expression of *PpABI3A* (Pp1s7_115V6), *PpABI3B* (Pp1s173_143V6.1) and *PpABI3C* (Pp1s143_82V6) in WT with or without cold acclimation for two weeks under 10°C. qRT-PCR analysis was used to monitor the expression of target genes. *PpACTIN5* (Pp1s381_21V6) was used as internal control. Error bars represent SD (n = 3) and Two-way ANOVA was used to determine the statistical significance (**, $P < 0.01$; ***, $P < 0.001$).
