# Supplementary material for: ABSCISIC ACID INSENSITIVE3 Is Involved in Cold Response and Freezing Tolerance Regulation in Physcomitrella patens
Source: Front Plant Sci. 2017 Sep 12;8:1599. doi: 10.3389/fpls.2017.01599 (PMC5601040; doi:10.3389/fpls.2017.01599)
Supplement: Supplementary file 4 [file Presentation2.PDF]

Figure S2

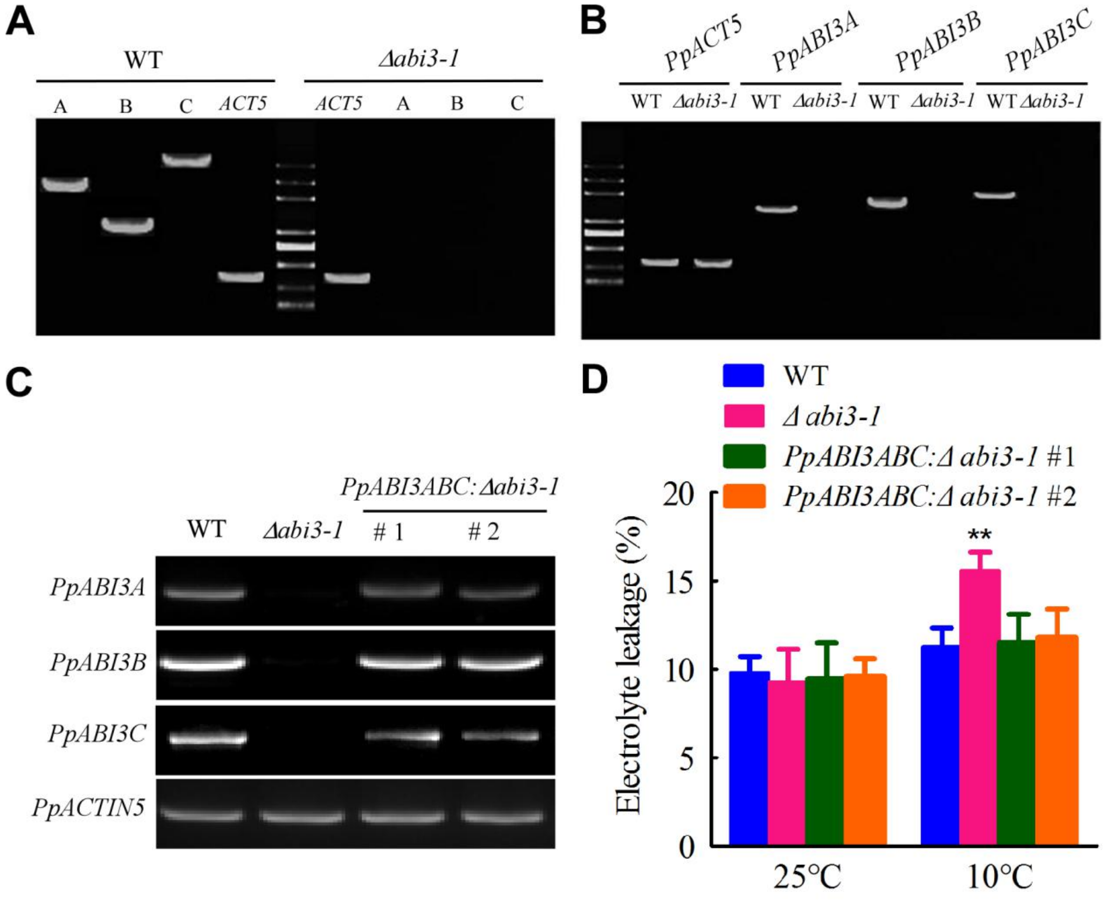

Figure S2. Identification of *Δabi3-1* mutant and complementary lines, and electrolyte leakage rate analysis.

(A). Genomic identification of *Δabi3-1*. DNA isolated from WT and *Δabi3-1* was used to determine the deletion of *PpABI3A*, *PpABI3B* and *PpABI3C* by PCR assays.

(B). RT-PCR assays for *Δabi3-1* mutant identification. Tissues of WT and *Δabi3-1* were collected for RNA isolation and reverse transcription PCR to detect the expression of each *PpABI3* gene.

(C). RT-PCR assays to detect the expression of three *PpABI3* genes (*PpABI3A*, *PpABI3B*, *PpABI3C*) in two complementary lines. Two-week-old tissues of *PpABI3ABC:Δabi3-1 #1* and *#2* were collected for RNA isolation and reverse transcription PCR. *PpACT5* was used as internal control, and specific primer sequences are listed in Table S1.

(D). EL rate analysis to estimate the cell viability. Tissues of WT, *Δabi3-1* and complementary lines after incubated under 25°C or 10°C were collected for EL measurement. Error bars represent SD (n = 3), two-way ANOVA was used to determine the statistical significance (**, $P < 0.01$).
