# Supplementary material for: ABSCISIC ACID INSENSITIVE3 Is Involved in Cold Response and Freezing Tolerance Regulation in Physcomitrella patens
Source: Front Plant Sci. 2017 Sep 12;8:1599. doi: 10.3389/fpls.2017.01599 (PMC5601040; doi:10.3389/fpls.2017.01599)
Supplement: Supplementary file 5 [file Presentation3.PDF]

Figure S3

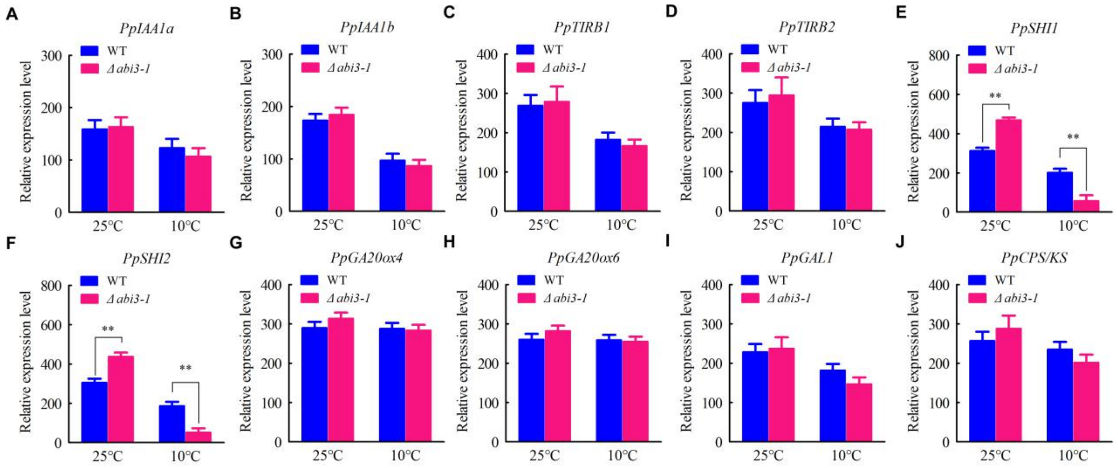

Figure S3. Quantitative-RT-PCR analysis of the transcription of representative auxin- or gibberellin- related genes.

(A)-(F). Expression of representative auxin-related genes. Representative auxin-related genes including *PpIAA1a* (Q948Q2), *PpIAA1b* (Pp1s184_21V6), *PpTIRB1* (Pp1s137_148V6), *PpTIRB2* (Pp1s196_87V6), *PpSHI1* (Pp1s373_11V6) and *PpSHI2* (Pp1s19_109V6) in WT with or without cold acclimation for two weeks under 10°C were quantified by qRT-PCR analysis. *PpACTIN5* (*ACT5*) was used as internal control. Error bars represent SD (n = 3) and Two-way ANOVA was used to determine the statistical significance (**, P < 0.01).

(H)-(J). Expression of representative gibberellin-related genes. Representative gibberellin-related genes including *PpGA20ox4* (Pp1s180_73V6), *PpGA20ox6* (Pp1s106_2V6), *PpGAL1* (PHYPADRAFT_235432) and *PpCPS/KS* (Pp1s130_5V6) in WT with or without cold acclimation for two weeks under 10°C were quantified by qRT-PCR analysis. *PpACTIN5* (Pp1s381_21V6) was used as internal control. Error bars represent SD (n = 3) and Two-way ANOVA was used to determine the statistical significance (**, P < 0.01).
