# Supplementary material for: ABSCISIC ACID INSENSITIVE3 Is Involved in Cold Response and Freezing Tolerance Regulation in Physcomitrella patens
Source: Front Plant Sci. 2017 Sep 12;8:1599. doi: 10.3389/fpls.2017.01599 (PMC5601040; doi:10.3389/fpls.2017.01599)
Supplement: Supplementary file 6 [file Presentation4.PDF]

Figure S4

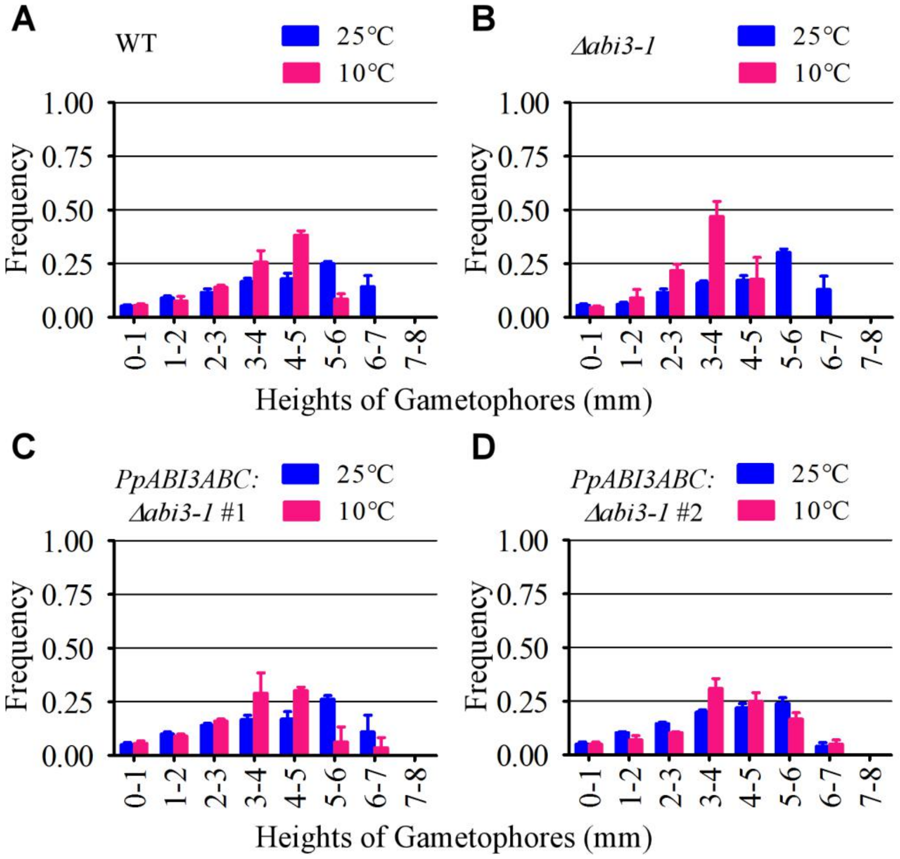

Figure S4. Frequency distribution of gametophore heights for the WT, *Δabi3-1* and *PpABI3ABC*:*Δabi3-1* lines.

(A)-(D). Seven-day-old tissues of WT, *Δabi3-1* and two complementary lines were inoculated on fresh BCD medium, and then grown under growth temperature (25°C) or lower temperature (10°C) for 4 weeks. Height of each gametophore per colony was determined and the distribution frequency was calculated.
