# Supplementary material for: ABSCISIC ACID INSENSITIVE3 Is Involved in Cold Response and Freezing Tolerance Regulation in Physcomitrella patens
Source: Front Plant Sci. 2017 Sep 12;8:1599. doi: 10.3389/fpls.2017.01599 (PMC5601040; doi:10.3389/fpls.2017.01599)
Supplement: Supplementary file 7 [file Presentation5.PDF]

Figure S5

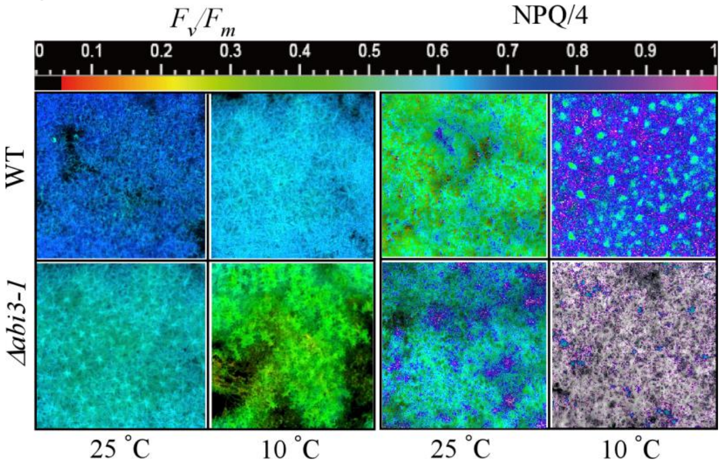

Figure S5. Determination of chlorophyll fluorescence.

Protonematal tissues of WT and *Δabi3-1*with or without cold acclimation for 2 weeks were collected to measure F_v/F_m and NPQ. Images of F_v/F_m and NPQ/4 were captured upon illumination for 30 s to 180 s. Signal intensities for F_v/F_m and NPQ/4 are indicated according to the colour scale (from 0 to 1.0) at the top.
